# Quantifying undetected tuberculosis in Ethiopia using a novel geospatial modelling approach

**DOI:** 10.1038/s41598-025-09171-z

**Published:** 2025-09-30

**Authors:** Haileab Fekadu Wolde, Archie C. A. Clements, Andargachew Kumsa Erena, Solomon Kassahun Gelaw, Samson Warkaye Lamma, Kefyalew Addis Alene

**Affiliations:** 1 School of Population Health, Faculty of Health Sciences, Curtin University, Bentley, WA Australia; 2 Geospatial and Tuberculosis Team, Telethon Kids Institute, Nedlands, WA Australia; 3 Institute of Public Health, College of Medicine and Health Sciences, University of Gondar, Gondar, Ethiopia; 4 Research and Enterprise, Queen’s University Belfast, Belfast, UK; 5 Ministry of Health Ethiopia/National TB, Leprosy and Lung Diseases Control Program/USAID LEAP Local, Addis Ababa, Ethiopia; 6 Strategic Affairs Executive Office, Ministry of Health-Ethiopia, Addis Ababa, Ethiopia; 7 Ethiopian Public Health Institute, National Data Management Center for Health, Addis Ababa, Ethiopia

**Keywords:** Undetected tuberculosis, Spatial distribution, Ethiopia, Diseases, Medical research

## Abstract

Tuberculosis (TB) is the leading infectious cause of death globally, with approximately three million cases remaining undetected, thereby contributing to community transmission. Understanding the spatial distribution of undetected TB in high-burden settings is critical for designing and implementing geographically targeted interventions for early detection and control. This study presents the first estimates of numbers of undetected TB cases in Ethiopia at national and local levels using novel geospatial method. We employed a Bayesian geostatistical modelling framework, incorporating national TB prevalence survey and TB notification data together with climatic and environmental variables, to estimate the number of undetected TB cases at district and national levels. Spatial clustering of undetected TB cases was assessed using Moran’s Index statistic and Local Indicator of Spatial Autocorrelation (LISA). A Bayesian Poisson regression model with conditional autoregressive (CAR) prior structure was developed to identify drivers of the clustering. We estimated a total of 51,041 undetected TB cases (95% CI: 50,599, 51,486) in Ethiopia, with the majority of these cases predicted in the Oromia region (20,440), Amhara region (9614), and South Ethiopia region (6061). Spatial clustering of undetected TB cases was observed in districts near the international borders, including the Ethiopia-Somalia and Ethiopia-Kenya border regions, as well as in several districts of Southern Ethiopia. The number of undetected TB cases was negatively associated with the proportion of the population with good mass media exposure (Incidence rate ratio (IRR): 0.67 95% CI: 0.56, 0.80) and the proportion of the population with high wealth index (IRR: 0.73, 95% CI: 0.60, 0.90). Our findings revealed a high burden of undetected TB in Ethiopia, with spatial clustering in border regions and areas with limited healthcare access. Targeted TB screening interventions to communities with low socioeconomic status along with improving mass media exposure in these regions, could significantly reduce the burden of undetected TB in Ethiopia.

## Introduction

Tuberculosis (TB) is the leading cause of death from an infectious disease globally, killing an estimated 1.4 million people in 2024^1^. Approximately 87% of the world’s TB cases occur in the 30 high TB burden countries^2^. Ethiopia is one of these countries with a TB incidence rate of 119 per 100,000 population^1,2^. The main prevention and control strategy for TB is early diagnosis and prompt commencement of appropriate treatment. However, despite significant progress in diagnosis and treatment, under-detection remains a significant problem in TB programs across several countries. Globally, approximately three million TB cases remain undetected each year due to underreporting and underdiagnosis, where individuals either do not seek healthcare or are not accurately diagnosed^3^. The magnitude of under-detection is particularly high in sub-Saharan African countries, including Ethiopia, where weak healthcare systems and poor infrastructure are common^4^. These undetected cases contribute to ongoing community transmission and pose substantial challenges to achieving the global End TB targets.

TB patients who are not diagnosed early and treated with appropriate TB medications often experience poor treatment outcomes and face an increased risk of developing drug-resistant TB, disability, and premature mortality^5,6^. Additionally, undetected TB cases can be significant sources of infection, continuously transmitting the disease within households and communities, which substantially contributes to the high incidence rates observed in developing countries, including Ethiopia^7,8^. A single untreated bacteriologically confirmed pulmonary TB case can transmit the disease to approximately 15 individuals annually and over 20 individuals throughout the natural course of untreated disease^9^. Passive case-finding is the primary approach currently used by Ethiopia’s national TB control program, relying heavily on patients seeking medical attention. However, active case finding is recommended as an important strategy to detect missing cases and break the cycle of community transmission, particularly in low-income and middle-income countries with a high burden of TB^10,11^. A study conducted in Ethiopia demonstrated a 50–70% increase in TB case detection due to the implementation of active case-finding strategies compared to routine case finding. However, due to resource constraints, it is not feasible to undertake active case finding throughout the entire country, requiring alternative strategies focused on high-risk areas^12,13^. By comparing the number of cases identified through both passive and active case-finding approaches, the difference provides an estimate of undetected TB cases, thereby informing targeted interventions in regions with the highest burden.

Estimating the number of undetected TB cases according to geographical location is fundamental for program planners and healthcare providers to implement, monitor and evaluate TB control and prevention efforts. Intensified efforts might be required in high-risk areas to improve access to diagnosis and treatment as well as to increase reporting of detected TB cases. Estimation of under-reporting and under-diagnosis has traditionally relied on expert opinion, often produced through regional workshops or country missions^14^. This subjective approach does not consider sub-national variation in levels of under-reporting. More robust estimates and a better understanding of how undetected patients may be spatially distributed are essential for prioritizing interventions to communities with the highest burden of undetected disease. While previous studies applied geospatial methods to map TB prevalence and notification rates^15,16^, to the best of our knowledge, no published research has examined the spatial distribution of undetected TB cases in Ethiopia or elsewhere. This study addresses this gap by estimating the number of undetected TB cases in Ethiopia using novel geospatial method and mapping their spatial distribution across the country.

## Methods

### Study setting and design

This nationwide study estimates the number of undetected TB cases in Ethiopia, the second-most populous country in Africa, with diverse geographical landscapes. Districts in Ethiopia are the decentralized administrative units responsible for health resource allocation decisions, and they served as the units of analysis for our study. Ethiopia is among the top 30 countries burdened by TB and TB/HIV, according to the WHO. Since 1997, Ethiopia has implemented the DOTS strategy, leading to the first combined Tuberculosis and Leprosy Prevention and Control Program manual. Following WHO guidelines, Ethiopia uses Xpert MTB/RIF assays, smear microscopy, and TB culture for diagnosis^17^. All hospitals and health centers in Ethiopia provide essential TB diagnostic services^18^.

### Data sources

We used a combination of data sources to estimate the number of undetected TB cases per district, which was the main outcome variable for our study.

#### TB prevalence data

We consider TB prevalence surveys to be an accurate measure of the actual burden of TB in the community. The main sources of these data were the Ethiopia national TB prevalence survey^19^ and articles published in peer-reviewed journals. The Ethiopia TB prevalence survey is the only existing nationally representative survey, conducted between 2010 and 2011 across all nine Regional States and two City Administrations (Addis Ababa and Dire Dawa) of Ethiopia. It comprised data from 85 geographic locations^19^. We complemented the national survey dataset with several sub-national surveys conducted in different parts of the country between 2002 and 2023. These sub-national surveys were obtained through a systematic literature review of biomedical databases, which identified all pertinent publications reporting TB prevalence in Ethiopia. The surveys used GeneXpert, smear microscopy, and culture to confirm the diagnosis of TB. Comprehensive details regarding the search methodology, inclusion and exclusion criteria, as well as extraction strategies employed for the systematic literature review, are published elsewhere^15,20^.

#### TB notification data

We obtained national TB notification data from the Health Management Information System (HMIS) of the Ethiopian Ministry of Health. We extracted monthly counts of TB case notifications by district for the period June 2019 to June 2021. We chose these reporting years due to their completeness and proximity to the data used to estimate TB prevalence. We include only case notifications for pulmonary TB among adults aged 15 years and above to match the population in TB prevalence surveys. Furthermore, we opted for a one-month duration of notification data, as the median duration for presumptive TB cases from the initial onset of symptoms to diagnosis of TB at a health facility is one month in Ethiopia^21^.

#### Covariates

Potential covariates with country-wide representative data available at high resolution and plausibly associated with low TB detection rate were obtained from satellite images and publicly available sources (Supplementary Table S1). The thirty-year average value of climatic variables, including temperature and precipitation, was acquired from the WorldClim website^22^. Altitude data were obtained from the Shuttle Radar Topography Mission (SRTM)^23^. Additionally, data regarding travel time to the nearest city and access to healthcare facilities in 2020 were obtained from the Malaria Atlas Project (MAP)^24^. We determined the population of adults aged 15 years and older in 2020 by utilizing high-resolution gridded population estimates from the WorldPop Project^25^. All the covariates were resampled and aligned to a spatial resolution of 1 km^2^^15^. Additionally, five covariates were constructed explicitly for this analysis based on their known association with TB under-detection and the availability of relevant data. These variables include wealth index, educational status, media exposure, as well as knowledge and attitudes towards TB, which were extracted from the 2011 and 2016 Ethiopian Demographic Health Survey (EDHS) datasets. Good media exposure was defined as being exposed to at least one form of mass media, such as newspapers, radio, or television, at least once a week. This variable was used as a proxy for health literacy since mass media is a good way of communicating health information. Additionally, high wealth index refers to the proportion of individuals who fall into the rich or richest wealth index categories. The definition of all the variables in the analysis can be found in Supplementary file 2. Since the EDHS provided individual-level data, we converted these data into raster files for the entire country using the Bayesian Kriging interpolation approach, a method previously validated with DHS data^26^. This approach allowed us to create spatially continuous surfaces by incorporating spatial autocorrelation and aligning the interpolated surfaces to the other covariate raster files (Supplementary file 3).

### Statistical analysis

**Fig. 1:**
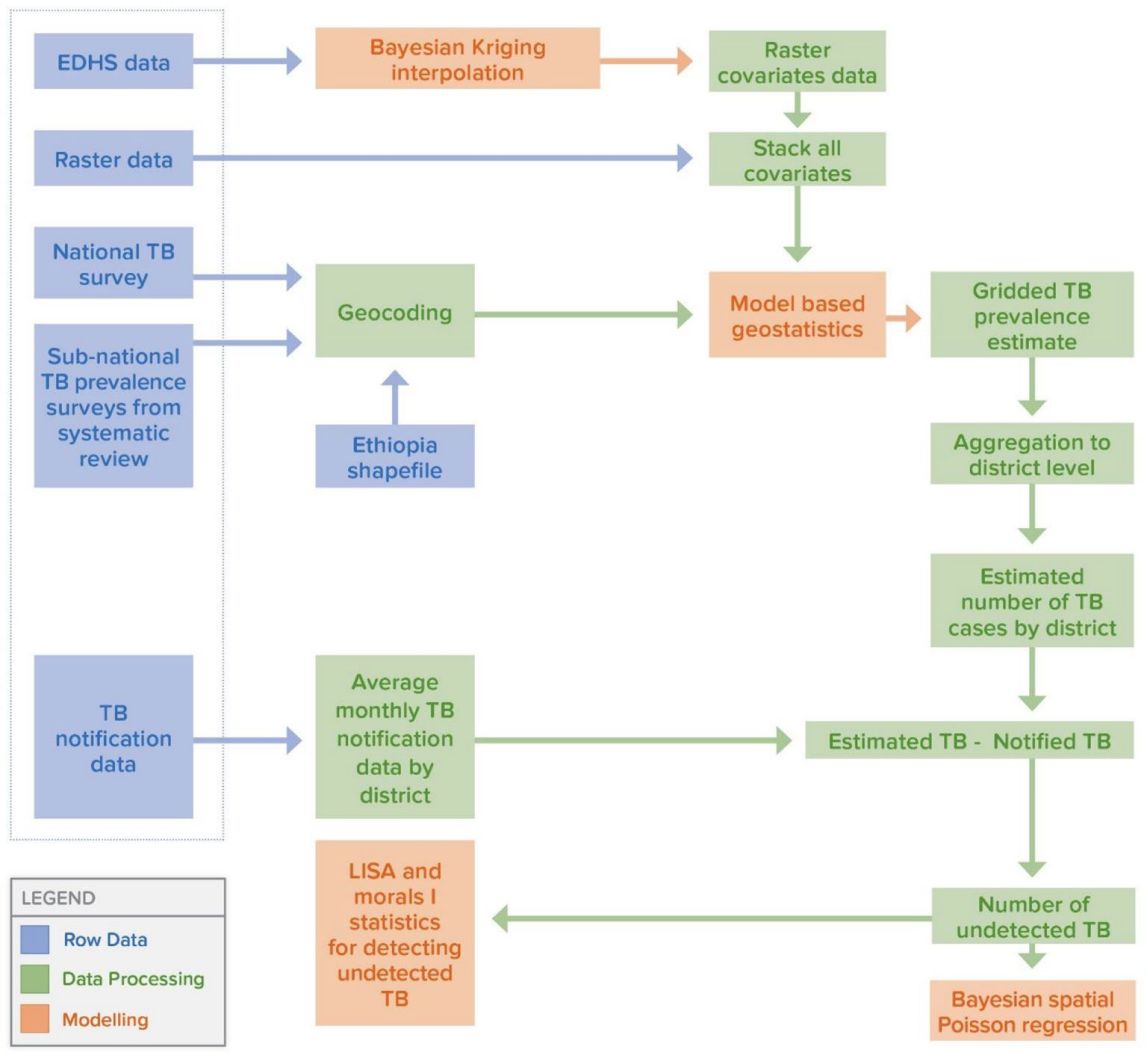
A flow diagram showing the data processing and modelling procedures used to estimate the number of undetected TB cases.

Figure 1 shows the procedures we followed to estimate the number of undetected TB cases at the district level across the country, using a Bayesian geospatial modelling approach.

#### Covariate stacking

We employed covariate stacking to align the covariates sourced from various datasets with different resolutions and extents. This method allowed us to integrate multiple raster datasets over the same spatial extent for spatial analysis. We performed cropping and masking of the raster files using the Ethiopian polygon shapefile and resampled the raster files to ensure that all covariates were standardized to the same resolution and spatial boundaries.

#### Geocoding of data

The TB prevalence and notification data were assigned a specific geographical location using ArcGIS Pro (ESRI, Redlands, CA). The Ethiopian Polygon Shapefile which contains the defined polygons representing administrative boundaries of districts within Ethiopia was used for geocoding. The geocoded data were linked to covariates. This involved integrating each TB prevalence and notification data point to the corresponding covariates in the same geographical area. This provided complete data for analysis that includes the outcome and associated covariates, all linked to specific geographic locations.

#### Geostatistical model

We fitted a Bayesian geospatial model to the TB prevalence survey data by incorporating covariates as fixed effects and the spatial component as a random effect using the Integrated Nested Laplace Approximation (INLA) approach implemented in R (R-INLA)^27,28^. The model was fitted using binomial logistic regression, assuming that the observed prevalence of TB ($${Y}_{i})$$ at location (*i*) follow a binomial distribution with a total number of people ($${n}_{i}$$) screened for TB and the predicted prevalence ($${p}_{i}$$) of TB at location $$i$$ (j = 1, …m): $${Y}_{i}\sim Binomial ({n}_{i},{p}_{i})$$;

The predicted TB prevalence was associated via a logit link function to a linear predictor defined as: $logit\left({p}_{j}\right)=\alpha + {\beta }_{j}*{{\varvec{X}}}_{j,i }{+ } {V}_{i}$

Here, $a$ is the intercept representing the overall risk of TB in the location $i$, ${\beta }_{j}$ is a matrix of covariate coefficients, ${{\varvec{X}}}$ is a matrix of $j$ covariates that were included in the model to quantify the source of variability at each location $i$. ${V}_{i}$ represent the spatial random effects that were modelled by a zero-mean Gaussian Markov random field (GMRF) with a Matern covariance function. The covariance function is defined by two parameters: the range *ρ*⁠, which represents the distance beyond which correlation becomes negligible (about 0.1 km), and *σ*⁠, which is the marginal standard deviation of the random field (in km)^29^. Non-informative Gaussian prior distributions with mean = 0 and precision (the inverse of variance) = 1 × 10^–4^ were used for both the intercept ($\alpha )$ and covariate coefficients (${\beta }_{j}$). The posterior mean, standard deviation, and 95% credible intervals were estimated for all the parameters.

From this geospatial analysis, we obtained maps for the predicted prevalence of TB at pixel level (1 km^2^). The number of TB cases was then determined by multiplying the estimated TB prevalence at each pixel by the corresponding at-risk population within that specific pixel, estimated from population density data. These estimated TB cases were then aggregated at the district level to obtain the number of TB cases, reflecting the true burden of TB in the community.

#### Undetected TB cases estimation

To estimate the number of undetected TB cases at the district level, we calculated the difference between the number of TB cases notified by the national surveillance system and the number of TB cases estimated from our geospatial modelling. The number of undetected TB cases in each district was georeferenced to map their spatial distribution, providing a visual representation of areas with undetected TB cases.

#### Hotspot analysis

Spatial clustering of undetected TB cases was explored at a global scale using Moran’s I statistic and at a local scale using Local Indicators of Spatial Association (LISA), estimated using the Anselin Local Moran’s I statistic, and the Getis-Ord statistic. The global Moran’s I statistic was used to assess the presence, strength, and direction of spatial autocorrelation in Ethiopia and to test the assumption of spatial independence in the implementation of the spatial pattern analysis^30^. The LISA and the Getis-Ord statistics were used to detect local clustering of undetected TB cases and to identify the locations of hot spots. These analyses were conducted using tools provided in ArcGIS Pro.

#### Spatial models for undetected TB cases

A Bayesian spatial Poisson regression model was constructed for the observed number of undetected TB cases (*Y*_*i*_) with a mean equal to the expected number of cases ($E{\text{i}}$) times the relative risk (*θ*_*i*_) corresponding to district $i, i=1,\dots ,n,$

$${\text{Y}}{\text{i}} \, \text{ Poisson(}E{\text{i}} \, \times \theta {\text{i}}\text{), }i=1,\dots ,n,$$

$$log \left(\theta {\text{i}}\right)=\alpha + \beta x + Ui + Vi.$$

Here, the logarithm of $\theta {\text{i}}$ was expressed as a sum of fixed effects to quantify the effects of the covariates on the undetected TB cases, and random effects that represent residual variation that is not explained by the available covariates. The fixed effects were expressed using a vector of intercept (*α)* and coefficients of the covariates $(\beta x).$ We included spatially structured random effects ($Ui$), which smooth data according to a predefined neighbourhood structure, to account for potential spatial autocorrelation (whereby the relative risks of undetected TB cases are likely to be more similar in neighbouring areas than in areas that are geographically distant). The spatial effects were structured using a conditional autoregressive (CAR) model. The neighbourhood matrix for the spatial random effects was defined such that two areas were considered neighbours if they shared a common boundary. Unstructured spatial components ($Vi$) were also included to model uncorrelated noise.

Due to the Bayesian characteristics of the geospatial model, priors needed to be defined for all parameters (and hyperparameters) in the model. Non-informative priors were used for *α* (uniform prior with bounds –∞ and ∞) and we set normal priors with mean = 0 and precision (the inverse of variance) = 1 × 10^–4^ for each ${\beta }_{j}$. The priors for the precision of the unstructured and spatially structured random effects were assigned non-informative gamma distributions with shape and scale parameters set at 0.001. Parameter estimation was done using the Integrated Nested Laplace Approximation (INLA) approach in R (R-INLA)^27,28^. Multiple variations of the model were fitted, and the best-fitting model was selected based on the deviance information criterion (DIC), whereby a model with a lower DIC value was selected as the better-fitting, more parsimonious model. All the analyses were conducted using R and ArcGIS software.

## Results

**Fig. 2:**
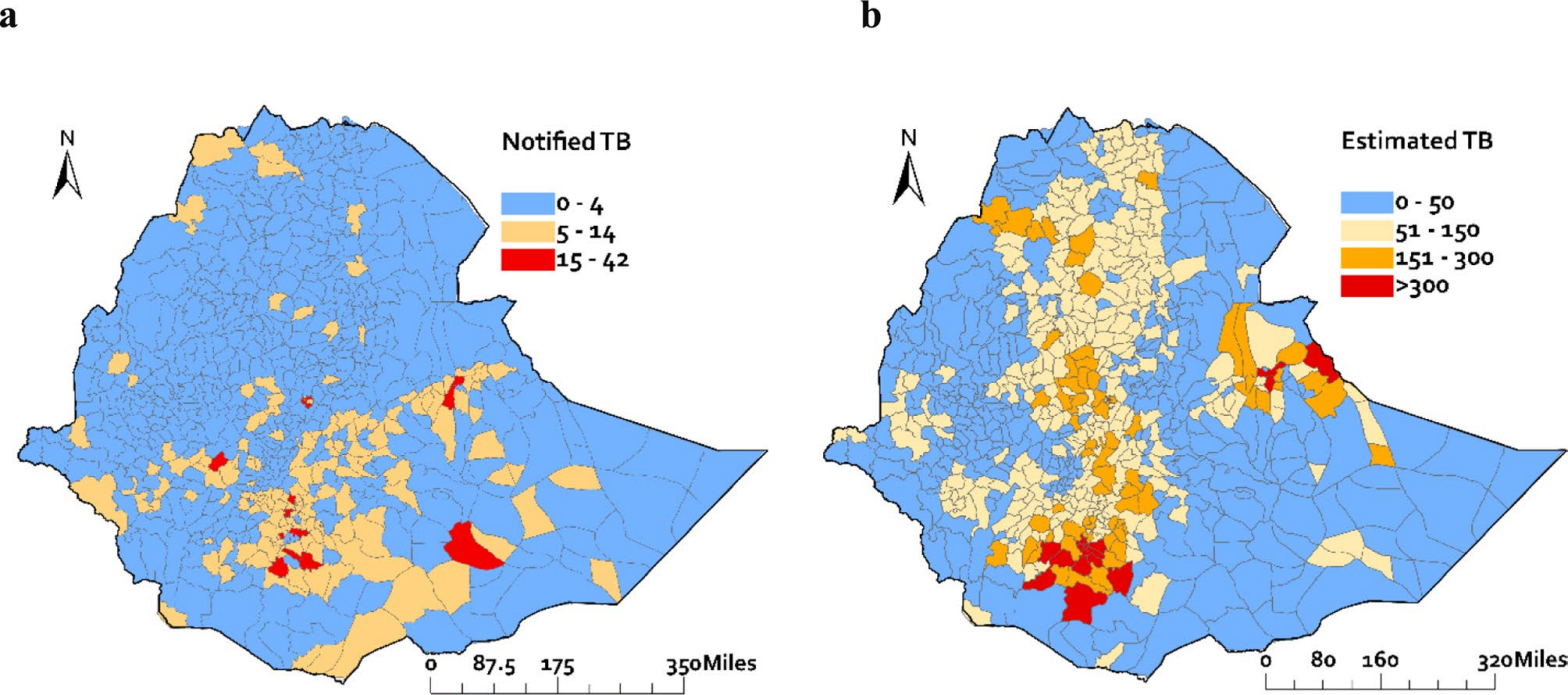
Number of notified tuberculosis cases reported by the national TB control program (**a**) and number of tuberculosis cases estimated by the geospatial model (**b**) at district level in Ethiopia.

Figure 2a shows the detected number of TB cases notified by the national TB control program in Ethiopia. The average number of monthly notified TB cases varies substantially across districts, ranging from 0 to 42 cases. Districts with high numbers of TB notifications were mainly observed in Oromia region, Sidama region, and Amhara region, while districts with low number of TB cases notifications were observed in Tigray and Amhara regions.

Figure 2b shows the number of estimated TB cases obtained from our geospatial modelling, representing the actual burden of TB cases in the district. High numbers of estimated TB cases were predominantly observed in regions including Oromia, South Ethiopia, Amhara, Addis Ababa, and Somali. The districts with the highest number of TB cases were Kochere Gedeb in Gedeo Zone of Southern Ethiopia Region, Lideta sub-city in Addis Ababa, and Dire Dawa city administration, with 474, 467, and 464 cases, respectively.

### Burden of undetected TB cases

Undetected TB cases were identified in 556 out of the 690 districts of the country included in our analysis, representing 80.6% of the total districts. The national estimation of undetected TB cases is 51,041 (95% CI: 50,599, 51,486). More than half of these undetected cases were concentrated in the Oromia and Amhara regions, followed by South Ethiopia region, while the Benshangul-Gumaz and Harari regions had the lowest numbers of undetected TB cases (Table 1).

**Table 1:** The estimated number of undetected TB cases by region in Ethiopia.

Regions	Number of undetected cases (95% CI)
Oromia	20,440 (20,161, 20,722)
Amhara	9614 (9423, 9808)
South Ethiopia	6061 (5908, 6214)
Addis Ababa	2436 (2340, 2535)
Sidama	2430 (2333, 2527)
Somali	2301 (2208, 2397)
Tigray	2250 (2158, 2345)
Central Ethiopia	2125 (2034, 2215)
Southwest Ethiopia	2058 (1969, 2147)
Dire Dawa	497 (454, 543)
Afar	340 (305, 378)
Gambela	309 (276, 345)
Harari	178 (153, 206)
Benshangul-Gumaz	2 (1, 7)
Total	51,041 (50,599, 51,486)

**Fig. 3:**
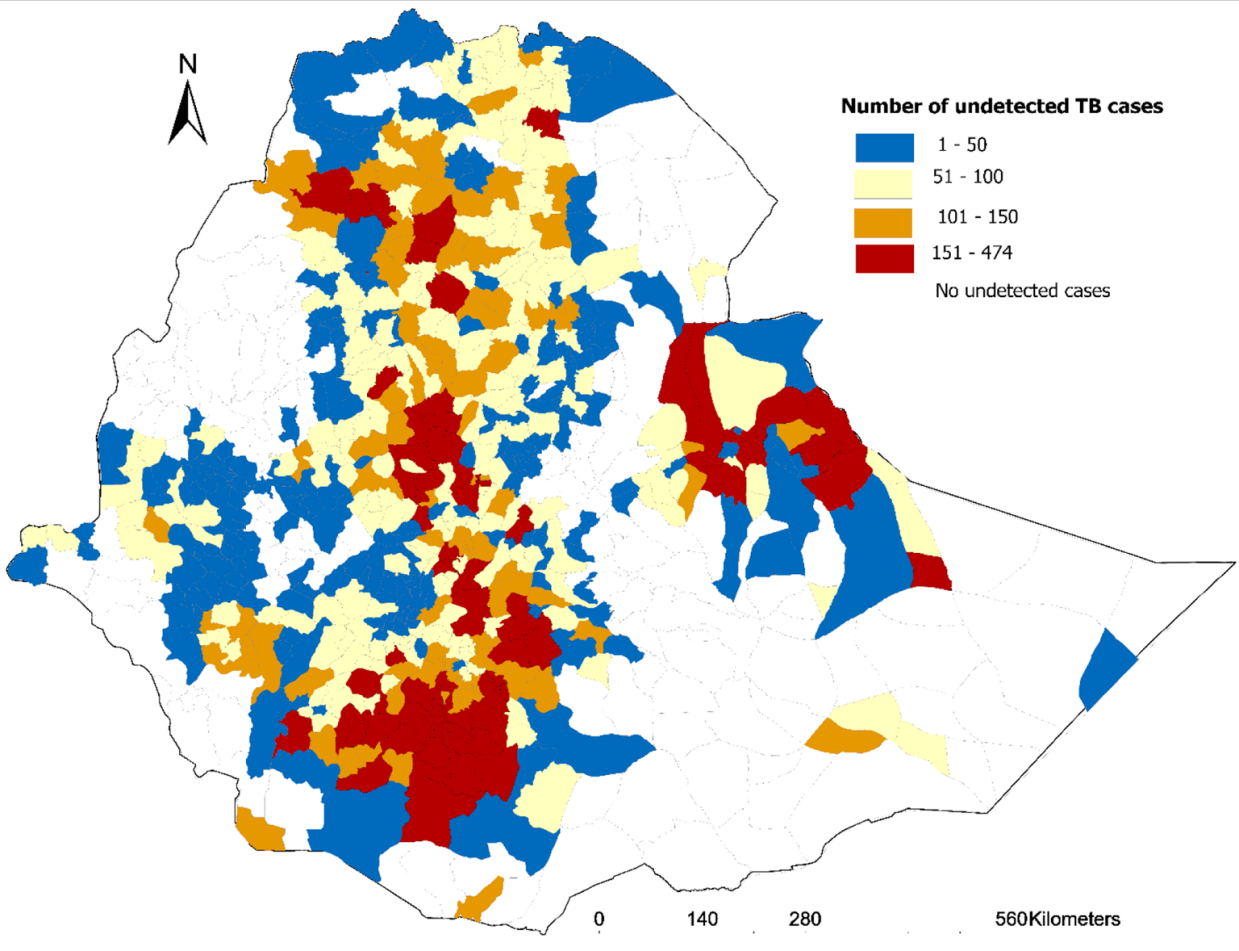
Estimated numbers of undetected tuberculosis cases at district level in Ethiopia.

Substantial spatial variations were noted in the number of undetected TB cases across districts in Ethiopia, ranging from 474 cases in the Kochere Gedeb district of South Ethiopia Region to 1 case reported in 11 districts spanning five regions. Districts with high numbers of undetected cases included Lideta sub-city in Addis Ababa, Asayita in Afar, Gondar Zuria in Amhara, Jikawo in Gambela, Bule Hora in Oromia, Aw-bare in Somali, and Enderta in Tigray (Fig. 3).

### Spatial clustering of undetected TB

**Fig. 4:**
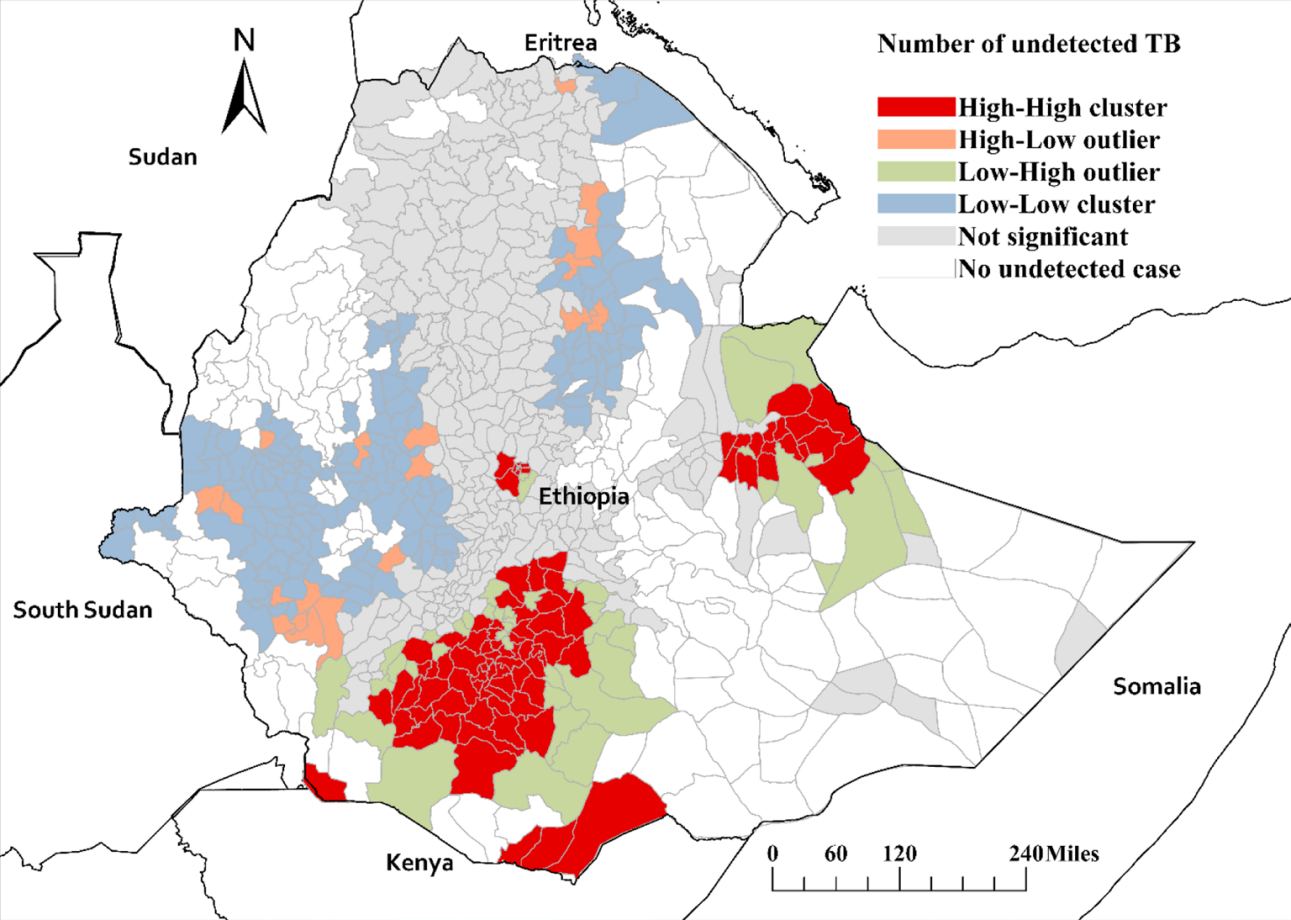
Spatial clustering of undetected tuberculosis in Ethiopia based on the Local Moran’s I statistic.

The spatial autocorrelation analysis, as determined by Global Moran’s I, yielded a statistically significant positive result (Moran’s I index = 0.20, p-value < 0.001), indicating spatial clustering of undetected TB cases across Ethiopia. Further analysis using Local Indicators of Spatial Association (LISA) revealed local clusters of high-high undetected TB cases in five districts of the Somali region, particularly prominent along international border areas with Somalia and Kenya. Additionally, high-high TB clusters were identified in all sub-cities of Addis Ababa, except Akaki-Kalite. Similar clustering patterns were observed in numerous districts within the East Harerge, West Arsi, and Guji zones of the Oromia region, as well as in a substantial number of districts within the South Ethiopia and Sidama regions. Conversely, low-low clusters of undetected TB cases were observed in districts within the Afar, Benshangul-Gumaz, Gambela, and Oromia regions**.** High-Low clusters were also identified in several districts across Ethiopia, including Kobo, Guba Lafto, and Kelela districts in Amhara; Guduru, Cheliya, Sayo, and Anfilo dstricts in Oromia; and Decha, Chena, South Bench, and Yeki in South Ethiopia. On the other hand, low- high clusters were identified in districts from Bale and Guji zones of Oromia region and other districts in southern Ethiopia (Fig. 4). The Getis-Ord statistics also showed a similar pattern of local clusters of undetected TB (Supplementary figure).

### Factors associated with undetected tuberculosis

Table 2 presents findings from a Bayesian multivariable Poisson regression model identifying ecological-level factors associated with the number of undetected TB cases. Results indicate that an increase in the proportion of individuals with a high wealth index decreased the number of undetected TB cases by 27% (IRR: 0.73, 95% CI: 0.60, 0.90). Additionally, the number of undetected TB cases decreased by 33% for a unit increase in the proportion of people with good media exposure (IRR: 0.67 95% CI: 0.56, 0.80).

**Table 2:** Bayesian spatial Poisson regression model for ecological-level factors associated with number of undetected TB cases in Ethiopia.

Variables	IRR (95%CrI)
High distance to a health facility	0.86 (0.71, 1.04)
Good knowledge of TB	0.78 (0.61, 1.02)
Good attitude towards TB	1.07 (0.88, 1.30)
High wealth index	0.73 (0.60, 0.90)*
Good media exposure	0.67 (0.56, 0.80)*
High education	1.10 (0.98, 1.24)
Intercept	3.02 (2.89, 3.14)

*IRR* incidence rate ratio, *TB* tuberculosis.

*Statistically significant variables.

**Fig. 5:**
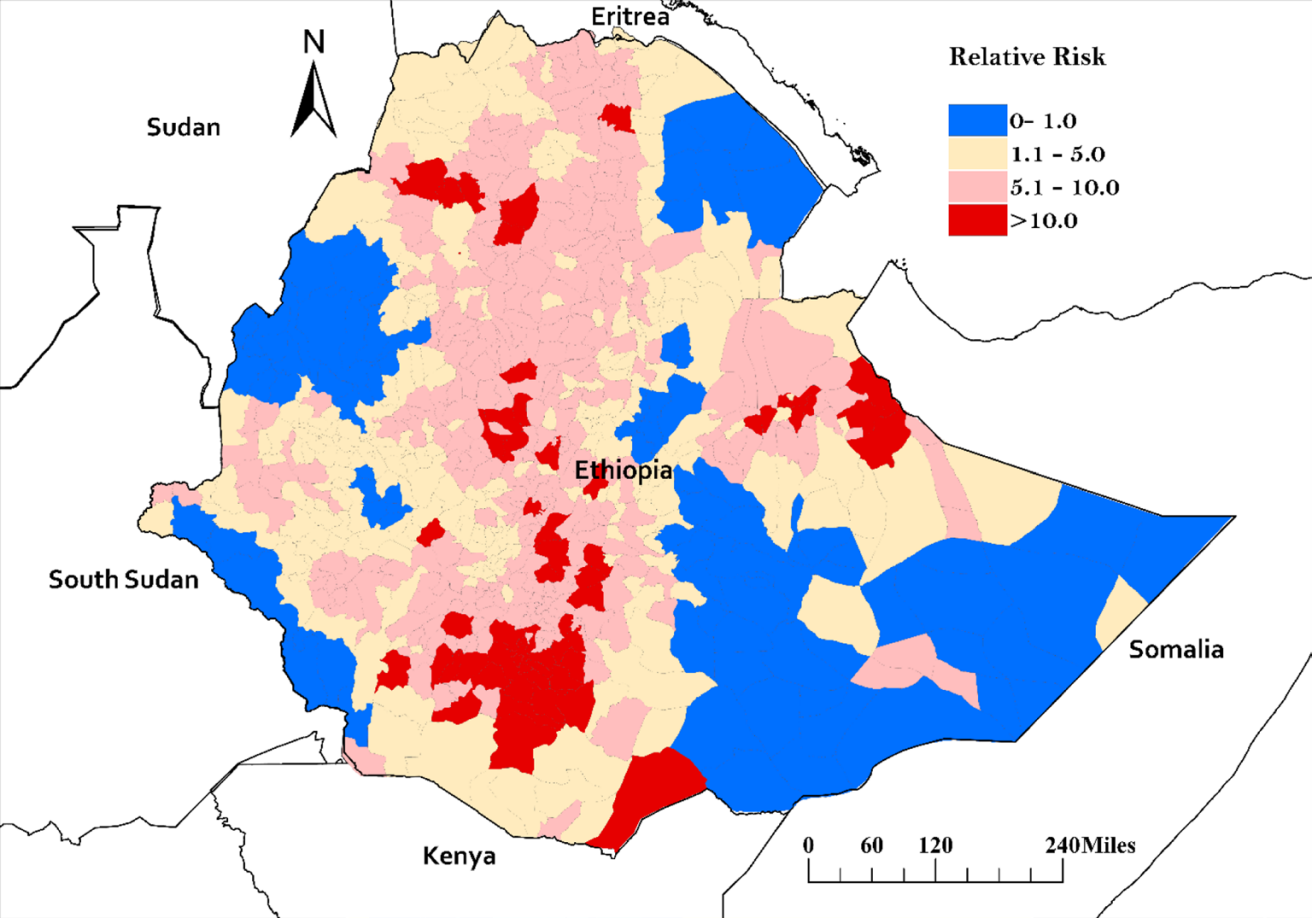
Map showing a district-level relative risk of undetected TB in Ethiopia estimated from spatial Poisson regression model.

The map of the posterior means of the relative risk after accounting for the model covariates demonstrated evidence of a high risk of undetected TB in several districts in Oromia, Somali, and South Ethiopia regions (Fig. 5).

## Discussion

In this study, we present the first nation-wide estimates of undetected TB cases across districts and regions of Ethiopia using advanced geospatial modelling approaches that incorporate TB prevalence surveys, notification data, and various covariates. Our findings reveal a high number of undetected TB cases at the national level, with substantial spatial variations at regional and local levels, which are associated with wealth index and mass media exposure.

The findings from our study reveal the critical challenge posed by undetected TB in Ethiopia, which remains a substantial barrier to TB control and elimination efforts. We estimated 51,041 undetected TB cases at the national level, which is similar to the WHO’s 2017 estimate of 55,275 cases that were either not diagnosed or not notified^31^. Clusters of high risk of undetected TB were observed in several districts of Southern Ethiopia, Addis Ababa and districts near the international borders, including Ethiopia-Somalia and Ethiopia-Kenya border areas which have been identified as areas of high TB transmission by a previous study^15^. Many of these districts in border areas are hard to reach, making it challenging for health services to reach everyone. There may also be a lack of adequate healthcare and diagnostic facilities equipped to detect TB. This is evidenced by a recent study showing that 65% of the population in the Somali region are unable to reach a health center or health post within one hour of walking, and no health center is adequately staffed according to national guidelines^32^. A high cluster of undetected TB was also observed in Addis Ababa and the Oromia region, including a substantial number of districts in the West Arsi Zone. Although there is better access to TB diagnostic and treatment services^33^, Addis Ababa is densely populated, which may facilitate high community-level transmission of the disease^34^. The city also attracts migrants from various areas of the country seeking employment, leading to overcrowded living situations and an increased risk of TB^35^. These marginalized communities often have poor access to health facilities for TB diagnosis due to poverty^35^ which could increase the burden of undetected TB^36,37^. Studies have also identified significant geographical clusters of high HIV burden in Addis Ababa and several districts in Oromia including the West Arsi zone^38^. This may contribute to an elevated number of TB cases in the community, as people living with HIV (PLHIV) face an 18-fold increased risk of developing TB, compared to their non-HIV counterparts^39^. Additionally, patients with HIV-TB co-infection often exhibit compromised immunity and reduced bacterial load in sputum, resulting in diminished detection rates using conventional TB diagnostic methods such as sputum smear, culture, tuberculin skin test, and interferon (IFN)-γ release test^36,39^, thereby potentially exacerbating underdiagnosis. This is supported by the WHO 2019 report, which indicated 44% of TB disease among PLHIV remain undiagnosed^40^.

This study found a negative association between high wealth status and the number of undetected TB cases, indicating that districts with a higher proportion of individuals with greater wealth exhibit lower numbers of undetected TB cases. This finding aligns with a global systematic review^41^ which identified financial barriers as a contributing factor to delayed TB diagnosis. Wealthier individuals are more inclined to seek prompt medical attention^42^ and have better access to TB diagnostic and treatment facilities^43^. Additionally, higher socioeconomic status is associated with enhanced living conditions, including improved housing quality, ventilation, and sanitation, all of which contribute to a reduced risk of TB transmission^44^. Furthermore, wealthier individuals often have access to a more diverse and nutritious diet, fostering a stronger immune system^45,46^.

Our study has identified media exposure as a significant factor in reducing the burden of undetected TB. Media platforms play a crucial role in disseminating information about TB symptoms, transmission, and available healthcare services, thereby empowering individuals to recognize symptoms early and seek prompt medical attention^43^, leading to improved detection of TB cases. In line with this, the WHO has emphasized communication through media as a key strategy to inform the public about available TB diagnostic and treatment services, as well as to educate individuals about the cardinal symptoms of TB, ultimately enhancing case detection and treatment outcomes^47^. However, the effect of media exposure should be understood within the context of socioeconomic disparities in Ethiopia. Access to media is often uneven, with wealthier and urban populations having better access to information through television, radio, and digital platforms, while rural and disadvantaged groups face limitations. Bridging this gap through targeted strategies like community radio and mobile interventions, and local health workers to disseminate information effectively, can enhance the reach of health messages, ensuring equitable improvements in TB detection^48^.

This study has some limitations. The accuracy of our estimates largely depended on the quality of HMIS data. Particularly noteworthy is the concern that the number of undetected TB in certain districts may be affected by the strength of their TB notification system. To overcome this limitation, we used the most recent three years of data reported to the Ministry of Health after the full implementation of the HMIS. While our study integrates diverse data sources to estimate the burden of undetected TB across districts in Ethiopia, some temporal inconsistencies between the covariates and the outcome data also remain a limitation. A spatiotemporal model will be required when new survey data become available, as the number of undetected TB cases may vary with time. This study focused on adults aged 15 years and older, due to a lack of TB prevalence data among children, limiting insights into the burden of undetected TB in this population.

## Conclusion

Our findings revealed a high burden of undetected TB in Ethiopia, with spatial clustering in border regions and areas with limited healthcare access, as well as the capital city. Targeted TB screening interventions to communities with low socioeconomic status along with improving mass media exposure in these regions, could significantly reduce the burden of undetected TB in Ethiopia.

## Supplementary Information

Supplementary Information.

## Data Availability

Data will be made available upon request to the corresponding author.
