# Supplementary material for: Quantifying undetected tuberculosis in Ethiopia using a novel geospatial modelling approach
Source: Sci Rep. 2025 Sep 30;15:33941. doi: 10.1038/s41598-025-09171-z (PMC12484886; doi:10.1038/s41598-025-09171-z)
Supplement: Supplementary file 1 — Supplementary Information. [file 41598_2025_9171_MOESM1_ESM.docx]

**Supplementary Materials**

Table of Contents

[Supplementary file 1: Definition of covariates used to map the prevalence of TB in Ethiopia 2](#_Toc170911692)

[Supplementary file 2: Definition of variables used to map district-level risk of undetected TB 2](#_Toc170911693)

[Supplementary file 3: Bayesian kriging interpolation 3](#_Toc170911694)

[Supplementary figure: Spatial clustering of undetected tuberculosis in Ethiopia based on the Getis-Ord statistics 5](#_Toc170911695)

# **Table S1:** Definition of covariates used to map the prevalence of TB in Ethiopia

| **Covariates** | **Data sources** | **Definitions** |
| --- | --- | --- |
| Population density | WorldPop | Number of people per square kilometre (grid) |
| Travel times to cities | Malaria Atlas Project (MAP) | Travel time in minutes to the nearest city with a population of more than 50,000 |
| Access to healthcare facilities | Malaria Atlas Project (MAP) | Walking travel times in minutes to the nearest health facility |
| Mean temperature | WorldClim | Annual mean air temperature in °C |
| Mean precipitation | WorldClim | Annual mean rainfall in mm |
| Altitude | Shuttle Radar Topography Mission (SRTM) | Elevation of the earth land surface in km |

# **Supplementary file 2:** Definition of variables used to map district-level risk of undetected TB

**Knowledge:** Data on the knowledge regarding TB were obtained from the EDHS 2011 survey(1). These data were collected by semi-structured questions by means of an interviewer-administered questionnaire. The TB knowledge of a person was assessed by three questions: 1) “have you ever heard of an illness called tuberculosis or TB (yes/no)”; 2) “how can a person get tuberculosis or TB?” and 3) “what symptoms will a person with tuberculosis or TB have?” A person was categorised as having “good” knowledge about TB if the person had ever heard about TB, correctly mentioned the route of transmission (i.e. TB is transmitted through the air when coughing or sneezing or through drinking of unboiled milk), and if the person mentioned at least one TB symptom (i.e. persistent cough for more than 2 weeks, weight loss, poor appetite, night sweats, chest pain or fever). Those who missed one or more of these three items were categorized as having ‘poor’ knowledge about TB.

**Attitude:** Data on the attitude regarding TB were obtained from the EDHS 2011 survey (1). The attitude of a person towards TB was measured by two questions: 1) can tuberculosis or TB be cured (yes, no, or don’t know) and 2) if a member of your family got tuberculosis or TB, would you want it to remain a secret? A “good” attitude was defined by a person believing that TB can be cured and not wanting a family member’s TB to be kept a secret. A “poor” attitude was defined by a person believing that TB cannot be cured or wanting a family member’s TB diagnosis to be kept a secret.

**Mass media exposure:** Data on the media exposure were obtained from the EDHS 2016 survey(2). This variable was used as a proxy for health literacy. Respondents were asked how often they read a newspaper, listened to the radio, or watched television. Those who responded at least once a week for any of the mass medias are considered to be regularly exposed to that form of media.

**Table S2:** summary of the Ecological level factors used to assess their effect on the number of undetected TB cases.

| **Independent variables** | **Data sources** | **Definition** |
| --- | --- | --- |
| Socio-economic factors | | |
| High wealth index | EDHS 2016 | Total number of people with high wealth index (rich and richest) divided by the total number of people participated in the survey. |
| Access to healthcare facilities | Malaria Atlas Project (MAP) | Walking travel times in minutes to the nearest health facility |
| Good media exposure | EDHS 2016 | Total number of people exposed to at least one of the media including newspaper, radio or television at least once a week divided by the total number of people participated in the survey |
| Good knowledge about TB | EDHS 2011 | Total number of people with good knowledge towards TB divided by the total number of people who participated in the survey. |
| Good attitude towards TB | EDHS 2011 | Total number of people with good attitude towards TB divided by the total number of people who participated in the survey |
| Better Educational status | EDHS 2016 | Total number of the population who had attended primary and above education divided by the total number of people who participated in the survey. |

# **Supplementary file 3:** Bayesian kriging interpolation

Baysian krining interpolation approach we used automatically calculates parameters through a process of subsetting and simulations and it also accounts for the error introduced by estimating the underlying semivariogram. The semivariogram parameters were estimated using restricted maximum likelihood (REML). Due to the computational limitations of REML for large datasets, the input data is first divided into overlapping subsets of 100 points. In each subset, first a semivariogram is estimated from the data in the subset then using this semivariogram as a model, new data is unconditionally simulated at each of the input locations in the subset. Then, a new semivariogram is estimated from the simulated data. This process created 100 simulated semivariograms per subset and each of these semivariograms is an estimate of the true semivariogram for the subset. For each prediction location, the prediction is calculated using a new empirical semivariogram distribution that is generated by merging the individual semivariograms from the semivariogram distributions in the point's neighborhood. Standard circular neighborhood searching was used with minimum and maximum neighborhood set to be 10 and 15, respectively. This prediction uses intrinsic random function as the kriging model and power semivariogram model.

#
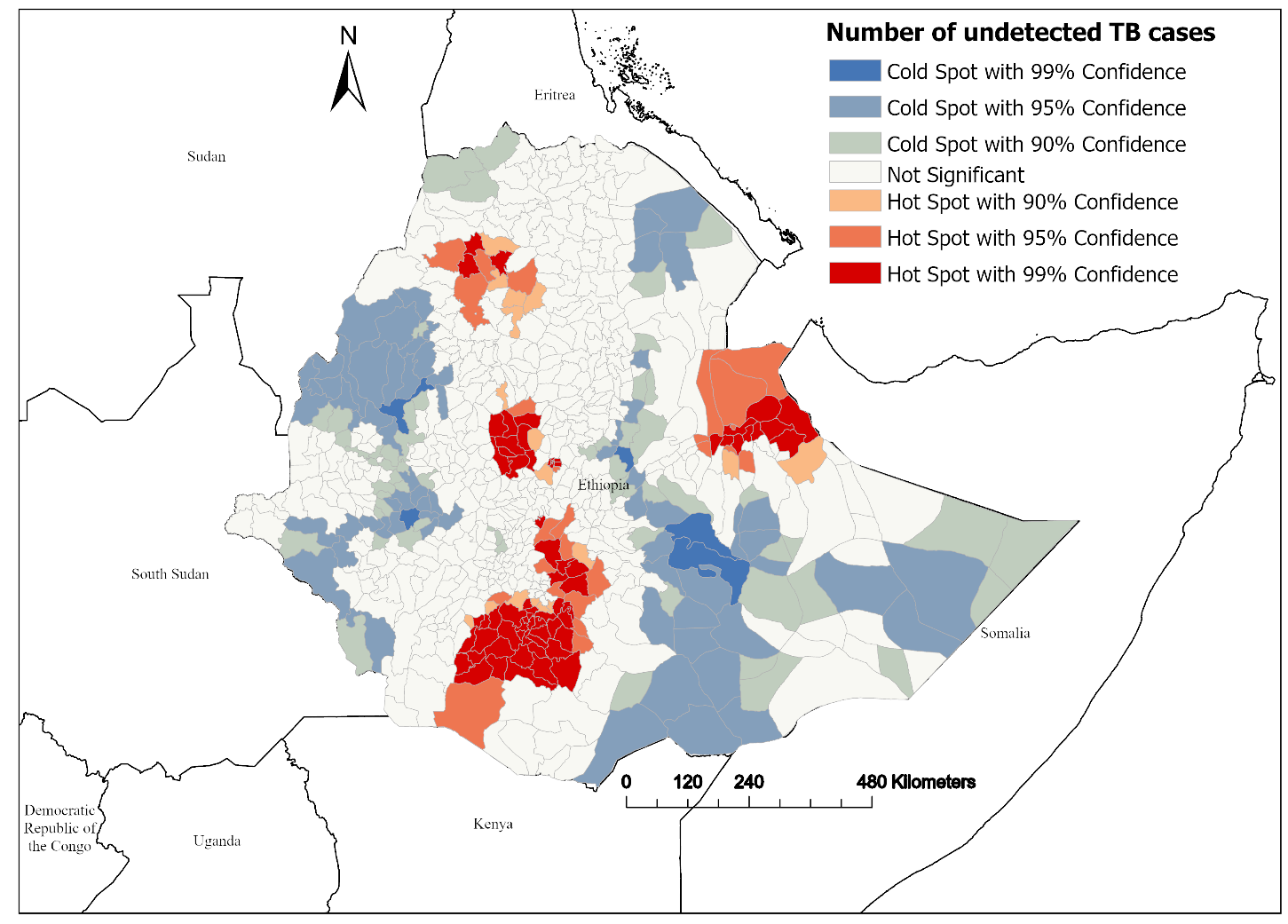
 **Supplementary figure:** Spatial clustering of undetected tuberculosis in Ethiopia based on the Getis-Ord statistics

**References**

1. CSA. In: International, editor. Ethiopia Demographic and Health Survey 2011. Addis Ababa, and Rockville: CSA and ICF: CSA; 2011

2. CSA. In: International, editor. Ethiopia Demographic and Health Survey 2016. Addis Ababa, and Rockville: CSA and ICF: CSA; 2016.
